# The complete evaluation of tube potential on clinical image quality when using direct digital detectors for pelvis and lumbar spine radiography: Re: ‘An evaluation of the effect of tube potential on clinical image quality using direct digital detectors for pelvis and lumbar spine radiographs’ by Peacock, Steward and Riley

**DOI:** 10.1002/jmrs.429

**Published:** 2020-12-04

**Authors:** Kholoud Alzyoud, Beverly Snaith, Peter Hogg, Andrew England

**Affiliations:** ^1^ Hashemite University Zarqa Jordan; ^2^ University of Bradford Bradford UK; ^3^ University of Salford Salford UK; ^4^ Keele University Staffordshire UK

We read with interest a recent submission to the *Journal of Medical Radiation Sciences* entitled ‘An evaluation of the effect of tube potential on clinical image quality using direct digital detectors for pelvis and lumbar spine radiographs’ by Peacock, Steward and Riley.^1^ We would like to thank the authors for their valuable contribution and acknowledge that they have attempted to assess an important issue and to provide further validation of high tube potential as a dose‐saving technique. Having previously worked in this area, we read this work with interest.

The authors discuss the benefit of high tube potential techniques but as with other studies do not provide a concise definition of ‘high tube potential’. Data presented in their study compare 75 and 85 kV for AP pelvis projections and 80 and 90 kV for lateral lumbar spine projections. We would argue that some authors would classify all of these tube potentials as ‘high’ or that ‘high kV’ would exist when using setting above or equal to 100 kV.^2^ It is also likely that the term high kV may have different means depending on the examination area, for example chest radiography. The authors accept that other tube potentials should be studied and that their work confirms several previously reported trends.^3^, ^4^

There are several methodological points we would like the authors to consider. A potential problem in this paper is that there are clearly a number of different image acquisition protocols in operation within the study institution, could the authors confirm why this is the case? A group of patients, presumably at the discretion of the operator, had acquisitions using 75/80 kV, and a further group had images acquired at 85/90 kV, for pelvis and lumbar spine radiography, respectively. We also suggest that post‐exposure mAs values cannot reliably conclude that the patients were comparable. Pathological variability can induce differences, for example using the central AEC chamber in the presence of scoliosis (Figure 1B). Also, when considering age‐related changes, such as osteoporosis, it could be possible for patients of a similar habitus but require very different exposure factors. It would be interesting to know why different tube potentials were in current clinical use, was this based solely on operator discretion, considering variables like patient size and age. Deviation Index (DI) also has widely reported frailties^5^ and as such does not necessarily imply either adequate exposure or image quality. Rather than standardising patient size between experimental groups, we believe it important to test a range of patient sizes which would be more clinically relevant.^4^ In doing so, it is important to understand whether optimisation methods, such as high tube potentials, work consistently across all patient sizes. With rising levels of obesity,^6^ this is likely for this issue to become even more important.

Surprisingly, there was a wide range in image quality reported in the study; for example, image quality ranged from 7 to 15 for pelvis images and 4 to 15 for lateral lumbar spine images. Would this variation in the 20 images not be a cause for concern, would the authors not expect less variation if automatic exposure control was used? It would have been useful if the authors had reported the variability between the senior radiographers in assessing image quality. Additionally, were there any other protocol variations, other than tube potential to consider?

We would also like to ask about the statistical analysis and presentation of results. Dose area product (DAP) is summarised as mean values yet there is no indication of spread (i.e. standard deviations); additionally, there are no inferential comparisons between dose values, despite this being performed for image quality. The authors may wish to supply such data within their response to this letter.

We would like to commend the authors on undertaking a clinical study to validate the effects of increasing tube potential. We strongly believe that variation in patient size must be included within such analyses and pathologies which will influence outcomes are recognised and included. We, the current authors and the wider research community must ensure that optimisation methods are fully inclusive and suggest that collaborative, multicentre evaluations are the key to building an evidence base for our profession. We acknowledge that authors have made comments regarding the future direction of their work and we look forward to seeing their results. Finally, we would be interested to know if the authors have made alterations to their local practice based on findings from their paper.

## Conflict of Interest

The authors declare no conflict of interest.
